# Designing and validating a PVA liver phantom with respiratory motion for needle-based interventions

**DOI:** 10.1007/s11548-019-02029-6

**Published:** 2019-07-11

**Authors:** Tonke L. de Jong, Adriaan Moelker, Jenny Dankelman, John J. van den Dobbelsteen

**Affiliations:** 1 BioMechanical Engineering Department, Delft University of Technology, Delft, The Netherlands; 2 Radiology and Nuclear Medicine Department, Erasmus MC, University Medical Center, Rotterdam, The Netherlands

**Keywords:** Liver phantom, Needle deflection, Interventional radiology, Respiratory motion

## Abstract

**Purpose:**

The purpose is to design and validate an anthropomorphic polyvinyl alcohol (PVA) liver phantom with respiratory motion to simulate needle-based interventions. Such a system can, for example, be used as a validation tool for novel needles.

**Methods:**

Image segmentations of CT scans of four patients during inspiration and expiration were used to measure liver and rib displacement. An anthropomorphic liver mold based on a CT scan was 3D printed and filled with 5% w/w PVA-to-water, undergoing two freeze–thaw cycles, in addition to a 3D-printed compliant rib cage. They were both held in place by a PVA abdominal phantom. A sinusoidal motion vector, based on the measured liver displacement, was applied to the liver phantom by means of a motion stage. Liver, rib cage and needle deflection were tracked by placing electromagnetic sensors on the phantom. Liver and rib cage phantom motion was validated by comparison with the CT images of the patients, whereas needle deflection was compared with the literature.

**Results:**

CT analysis showed that from the state of expiration to inspiration, the livers moved predominantly toward the right (mean: 2 mm, range: − 11 to 11 mm), anterior (mean: 15 mm, range: 9–21 mm) and caudal (mean: 16 mm, range: 6–24 mm) direction. The mechatronic design of the liver phantom gives the freedom to set direction and amplitude of the motion and was able to mimic the direction of liver motion of one patient. Needle deflection inside the phantom increased from 1.6 to 3.8 mm from the initial expiration state to inspiration.

**Conclusions:**

The developed liver phantom allows for applying different motion patterns and shapes/sizes and thus allows for patient-specific simulation of needle-based interventions. Moreover, it is able to mimic appropriate respiratory motion and needle deflection as observed in patients.

**Electronic supplementary material:**

The online version of this article (10.1007/s11548-019-02029-6) contains supplementary material, which is available to authorized users.

## Introduction

Research indicated that 90% of interventional radiologists believe that reachability of the lesion is challenged by target movement due to breathing of the patient [1]. Moreover, it is indicated that liver and rib motion due to breathing is one of the factors that contribute to unwanted needle deflection upon insertion. During radiofrequency ablations of liver tumors, needle deflection of several mm is encountered [2], thereby increasing the total targeting error. These studies indicate the importance of including proper liver and rib motion in a liver phantom for needle-based interventions.

Current developments in medical robotics and novel instrument design have increased the demand for physical validation setups. For example, Van de Berg et al. [3] presented a manually steerable needle to be used in interventional radiology and studied its endpoint accuracy in homogeneous gelatin phantom blocks. Preferably, these prototypes would be tested in vivo, i.e., in human patients. However, due to safety and ethical reasons, this is not a feasible option. Therefore, realistic alternatives are needed, such as tissue-mimicking phantoms that are able to mimic the heterogeneous features of real tissue.

The importance of certain phantom requirements depends on its specific application area, as emphasized by an extensive review on tissue-mimicking materials by Li et al. [4]. In the present study, we focus on the development of a liver phantom for needle-based interventions. For this specific case, it requires: (1) a phantom material with matching *needle*–*tissue interaction forces* and feasibility to be used with *ultrasound,* (2) an *anthropomorphic* phantom shape and (3) liver and rib *motion* subject to respiration of the patient.

The aforementioned phantom requirements have been studied to a greater or lesser extent. Recent studies indicate the suitability of polyvinyl alcohol (PVA), a polymer that forms molecular cross-links upon freezing and thawing, in matching the needle–tissue interaction forces of human liver [5, 6]. This material resembles the heterogenic structure of real tissue, in contrary to many other homogeneous base materials, such as gelatin [7], PVC [8] and agarose [9]. In addition, PVA has good ultrasound-mimicking properties, as shown by several studies, e.g., [10, 11].

Other research mimicked the anthropomorphic shape of the liver. For example, Rethy et al. [12] developed a multimodal permanent liver phantom displaying functional vasculature and pathologies, by wax and silicone molding of a donated human liver. In addition, Efthymios et al. [13] presented anatomically realistic ultrasound phantoms based on patient scans, by pouring gel wax in 3D-printed molds.

The third requirement, liver and rib motion due to breathing of the patient, is studied to lesser extent. During breathing, the liver moves not only with respect to the static world, but also with respect to ribs and skin. Several attempts have been made to include this motion in a liver phantom. The first one dates back to 1968 and was described by Stewart et al. [14]. This basic phantom, made from a plastic container and paraffin, simulates breathing by applying longitudinal travel with an amplitude of 3 cm. Cleary et al. [15, 16] developed a liver respiratory motion simulator, by connecting a human torso and liver model to a linear motion platform at the base of the torso’s right abdomen. In addition, a recent paper by Naghibi et al. [17] presented the development of a soft robotic phantom to simulate the dynamic respirator motion of human liver, thereby focusing on MR compatibility. They used pneumatic soft actuators to generate motion patterns resembling those described in the literature [18, 19]. However, none of these phantoms include a combination of liver and rib motion, and they were not tested for corresponding needle deflection upon insertion.

In short, with the increasing demand for realistic phantoms for needle-based interventions, several studies have been performed. However, there are no phantoms available that fulfill all of the aforementioned requirements in a single setup. Therefore, the goal of the current study is designing and validating an anthropomorphic PVA liver phantom with respiratory motion that can be used for needle-based interventions.

## Approach

**Fig. 1:**
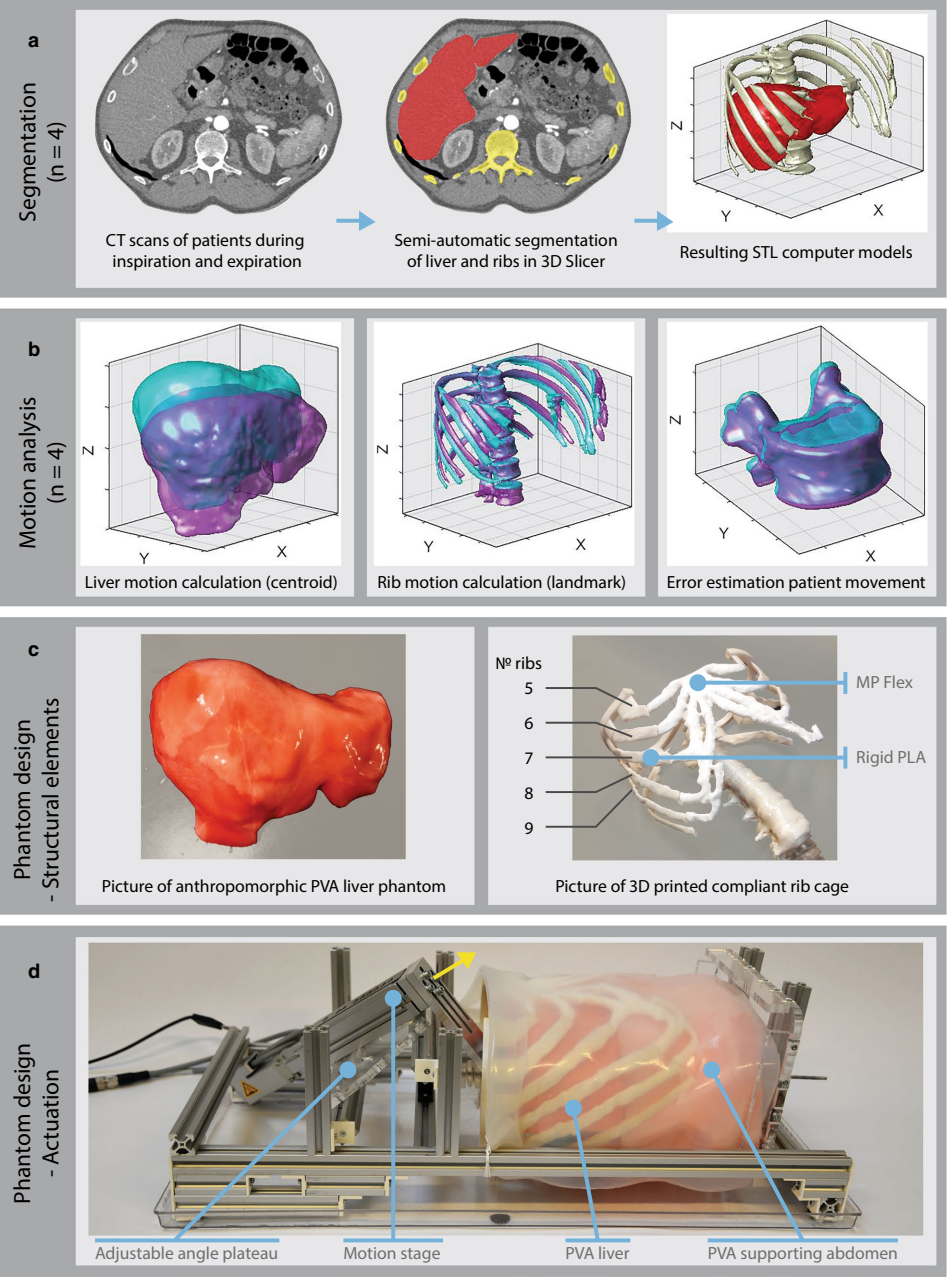
Process of the liver phantom design divided into four parts. **a** Segmentation of livers and ribs of the CT scans of five patients during inspiration and expiration. Resulting STL models were post-processed in MeshLab. **b** Motion analysis of the livers, ribs and an error estimation of patient movement during image acquisition. Blue = expiration, purple = inspiration. **c** The structural components of the phantom model, consisting of PVA liver phantom and 3D-printed ribs. **d** Actuation of the liver phantom using a motion stage

Four steps were defined in the research process that are illustrated in Fig. 1. First, we semiautomatically segmented the liver and rib cage of four patients using CT images during inspiration and expiration. Second, we quantified the liver and rib motion by calculating motion vectors. In addition, we estimated the error of patient movement during the two respiratory phases. Third, we designed the liver phantom, rib cage and actuation system, divided into structural elements and actuation. Finally, we performed experiments with an electromagnetic tracking system to track the liver phantom’s motion and needle bending. Liver phantom’s motion was validated with the data from the CT images, whereas needle bending was compared with data that have been described in the previous literature [2].

## Methods

### Liver and rib cage segmentation

The study population included patients that underwent computed tomographic (CT) angiography in both inspiration and expiration and were suspected to have mesenteric ischemia because of arcuate ligament syndrome. Patient data were retrospectively acquired from the hospital information system and Picture Archiving Communication System. The total dataset consisted of 40 patients, of which four were included in the analysis, because their scans contained the total liver volume and rib cage during inspiration and expiration. The data were processed and stored anonymously. The medical research ethics committee of the Erasmus University MC approved that the Medical Research Involving Human Subjects Act did not apply to this study and that no informed consent was required according to the local directives for retrospective studies (MEC-2016-241).

Semiautomatic segmentation of the livers and ribs, illustrated in Fig. 1a, was done with 3D slicer 4.8.1 [20] (http://www.slicer.org), using the following segment editor modules. “Draw” and “paint” were used for the manual part of the segmentation, and “Grow from seeds” was used for the automatic part. The last module applies a fast region growing method, in which final segment boundaries are placed where its master volume changes brightness abruptly, as described in [21]. This method turned out to be suitable for the rib cage, as brightness differences with the surroundings are high. However, in case of the livers, additional smoothing was required.

**Fig. 2:**
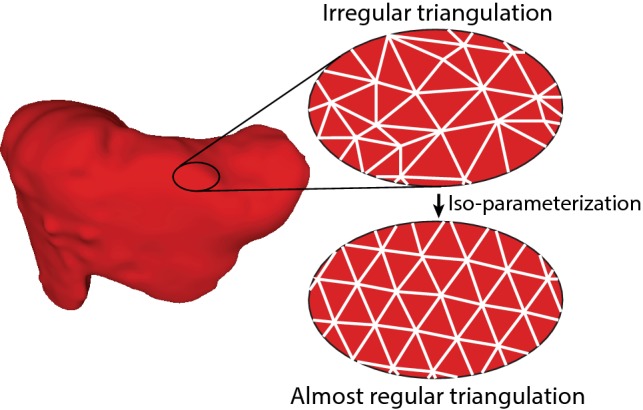
Post-processing of the STL models in MeshLab. The irregular triangulation pattern was iso-parameterized to an almost regular triangulation using [21]

The resulting models were stored as .stl files and loaded into MeshLab, an open source system for processing and editing 3D triangular meshes [22]. Several simplification, smoothing and re-meshing steps were performed. First, the models were cleaned by removing unreferenced and non-manifold vertices. Second, the Screened Poisson Surface reconstruction was completed to create a closed surface. Then, the models were simplified to 50,000 faces with MeshLab’s Quadric Edge Collapse Decimation, resulting in significant smaller file sizes (from ~ 42 to 2.5 mb) without losing their original shape. Next, Laplacian smoothing was performed, which calculates for each vertex the average position with the nearest vertex. Finally, iso-parameterization was executed for the liver models, as described by Pietroni et al. [23] (Fig. 2), to re-mesh the surfaces to almost regular triangulation with minimal distortion. The resulting files are *Open Access* and can be found online at [24]. The livers measured on average 198 mm in left–right (range: 180–223 mm), 160 mm in posterior-anterior (range: 119–200 mm) and 161 mm in cranio-caudal (range: 142–178 mm) view.

### Liver and rib cage motion analysis

#### Liver and rib motion

Liver movement was quantified in terms of translational motion, which we defined as the difference between the centers of mass during inspiration and expiration, thereby assuming uniform density of the liver tissue. In addition, rib motion was quantified by calculating the differences between inspiration and expiration at the transition point between bone and cartilage of the seventh rib.

To perform the analysis, the .stl files of the livers and ribs were loaded into MATLAB 2017b (The MathWorks, Inc., Natick, Massachusetts, United States). The iso-parameterization, described in the previous segmentation section, allowed for the calculation of the center of mass to quantify liver motion, as the distances between vertices are equally spaced. The following formula was used to compute the center:

$$ C_{x,y,z} = \mathop \sum \limits_{i = 1}^{n} \frac{{V_{{x_{i} y_{i} z_{i} }} }}{n} $$

with *C* is the position of the centroid, $V_{{x_{i} y_{i} z_{i} }}$ is the local position of the vertex and *n* is the total number of vertices.

#### Error estimation patient movement

An error estimation of patient movement during the inspiration and expiration phase was performed. This gives an estimate of the error in the quantification of liver and rib motion. We calculated the centroid of the ninth thoracic vertebras and compared the position in 3D space during both respiratory phases. Errors were given as the mean difference between the two states and standard deviation for the four patients.

### Liver phantom design

The developed phantom is a real size, anthropomorphic liver phantom made of PVA and consists of structural components of the liver itself and its actuation. The structural elements of the phantom are shown in Fig. 1c, whereas its actuation is depicted in Fig. 1d. A video of the phantom can be found online as electronic supplementary material.

#### Structural elements

The structural elements of the liver phantom consisted of a PVA liver, a support PVA abdominal cavity, a rib cage phantom and a skin phantom made of a 2.5-mm layer of silicone (Ecoflex 00-30, Smooth-on Inc., Macungie, Texas, USA). The PVA liver (Fig. 1c, left) was made by pour-molding PVA in a 3D-printed liver mold. This mold was created from the negative imprint of the computer liver model and 3D printed in polylactic acid (PLA) with 0.25 mm printing resolution. Subsequently, it was filled with 5% w/w PVA-to-water (Selvol PVOH165, Sekisui Chemical Group, NJ, USA), undergoing two freeze–thaw cycles for 72 h each at a temperature of around − 18 °C. The abdominal cavity phantom was also made of PVA (4% w/w PVA-to-water, two freeze–thaw cycles, 72 h each) and used to support the PVA liver. It was made by filling a box with PVA and used a 3D-printed liver as negative shape, which was removed after curing of the PVA. The concentrations for the PVA liver and abdominal cavity phantom were chosen based on previous research. This study characterized the needle–tissue interaction forces in healthy human liver and PVA phantoms in terms of magnitude of the needle insertion forces, number of force peaks and friction along the needle shaft [6]. We chose a higher concentration for the PVA liver (5% w/w) as compared to the abdominal cavity phantom (4% w/w) to simulate the stiffness of diseased liver tissue as compared to its less stiff, healthy abdominal surroundings. All PVA components were colored with food color.

The phantom ribs were 3D printed using two materials. The bony structures of the ribs and rib cage were printed using rigid PLA (MakerPoint, Digital Fabrication Center, the Netherlands), whereas the cartilage was printed with FLEX45, a flexible material (MakerPoint, Digital Fabrication Center, the Netherlands), to allow for compliant motion. These parts were glued together (Fig. 1c, right). An Ultimaker 3 (Ultimaker B.V., Geldermalsen, the Netherlands) was used for all 3D prints.

#### Actuation

The phantom liver was actuated by an EGSL-BS-35-50-8P mini-motion stage, with a maximal travel distance of 50 mm and minimum and maximum sliding velocities of 0.13 mm/s and 350 mm/s, respectively (Fig. 1d). It was powered by a EMMS-ST-28-L-SE stepper motor without brake. The stepper motor was controlled by a CMMS-ST-C8-7-G2 motor controller (all components from Festo BV, Delft, the Netherlands), which, in turn, was directed by a LabJack T7 (LabJack Corporation, Lakewood, USA).

### Motion validation of the liver phantom

**Fig. 3:**
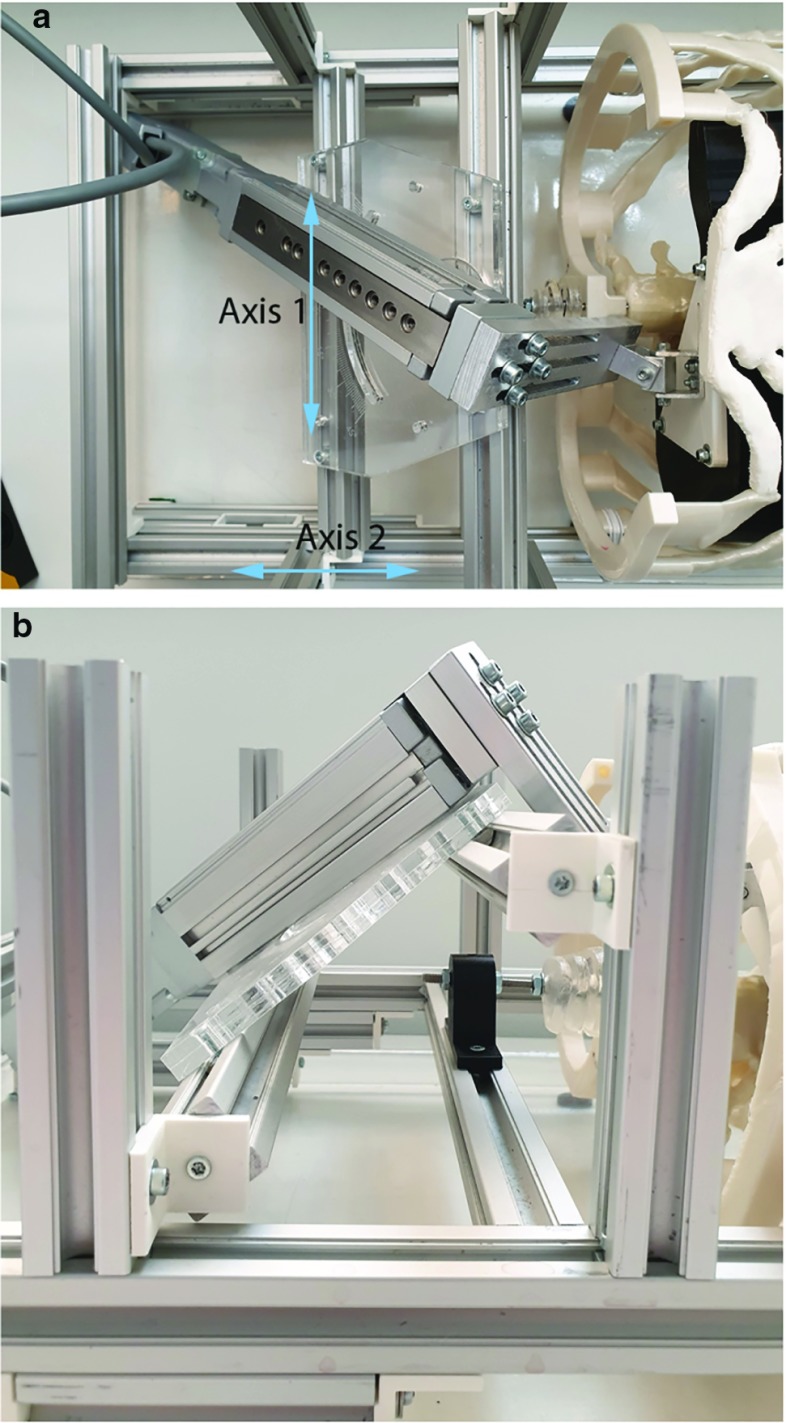
Adjustable direction of the linear stage to simulate breathing motion: **a** top view, indicating the left–right axis (axis 1) and forward–backward axis (axis 2) and **b** side view

The liver phantom motion was mimicked using the data of one patient. The generated motion pattern was a sinusoid (frequency: 12 Hz, position controlled), based on the measured liver displacement during breathing. A close-up of the linear stage is given in Fig. 3. The direction of breathing motion is adjustable by setting two angles of rotation. By tuning the motion stage around the left–right axis (axis 1) and the forward–backward axis (axis 2), the direction of the motion vector can be set as desired, based on the calculated position of the centers of the liver on the CT scans of a specific patient. The direction and amplitude of the motion vector were based on the results of the liver and rib cage motion analysis of the four patients and are therefore given in Results section. Motion was applied directly to the superior part of the PVA liver.

**Fig. 4:**
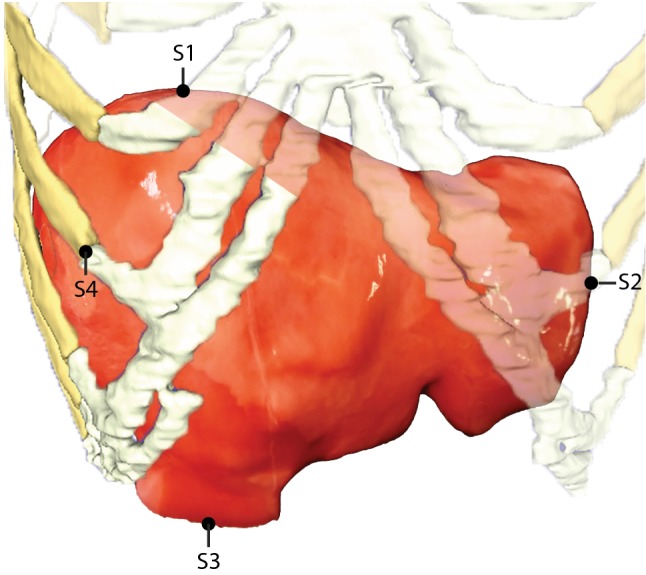
Sensors 1–3 were placed inside the PVA liver. Sensor 4 was placed on the transition between bone and cartilage of the seventh rib. A reference sensor was placed on the moving part of the linear stage

Motion of the liver phantom was validated using NDI Aurora’s Tabletop Field Generator (Northern Digital Inc, Ontario, Canada). This system generates an electromagnetic field in which sensors can be tracked real time in 3D space. Standard 0.8 mm Aurora 5DOF sensors were used (Part Number: NDI 610090). In total, three sensors were placed inside the PVA liver, and one on the rib cage (Fig. 4). One reference sensor was placed on the moving part of the linear stage. First, the noise levels were measured for 10 s with the motor switched on and off. Second, the phantom liver was actuated by the stage for 60 s, while capturing the motion of the different sensors. Then, noise levels were again measured for 10 s. This was repeated three times. Noise levels were expressed as a combined standard uncertainty, calculated by taking the root sum of the squares of the standard deviations of the measured positions of the reference sensor in 3D space.

**Fig. 5:**
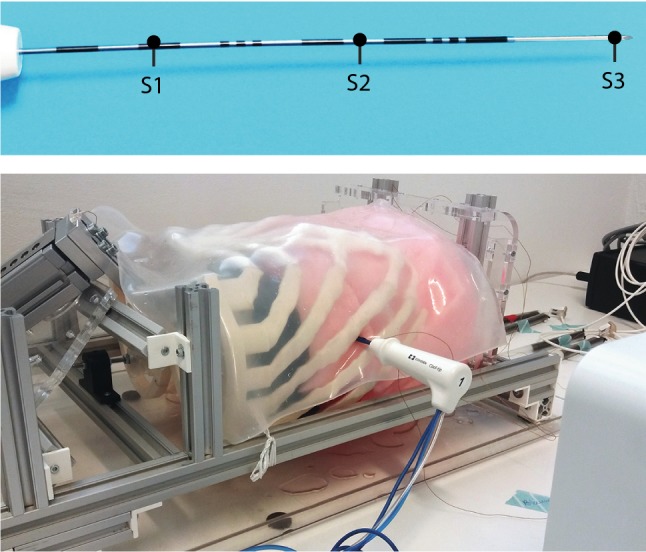
Three sensors were placed on the ablation needle to quantify deflection (upper part). A reference sensor was placed on the moving part of the linear stage. The needle was inserted between the eighth and ninth rib (lower part)

In addition to the validation of system’s motion itself, the deflection of a cool-tip ablation needle (17Gauge Cool-tip RF ablation system E, Medtronic, Minneapolis, USA) was measured for 60 s by inserting the needle approximately 10 cm into phantom between the eighth and ninth rib. The needle was inserted in expiration phase, i.e., in the initial position of the stage without motion. It had three sensors on it: one at the entry point of the liver phantom, one at the needle tip and one halfway the insertion (Fig. 5). The sensors were fixed onto the needle with isolation tape. Again, a reference sensor was placed on the moving part of the linear stage. Needle deflection was defined by calculating the point-to-line distance, with the point being the position of sensor 2 and the line being interpolated from the positions of sensors 1 and 3.

### Needle visibility: ultrasound

The liver phantom was checked for needle visibility by inserting the cool-tip ablation needle between the seventh and eighth rib under ultrasound imaging. An iU22 Ultrasound Machine (Philips Medical Systems International B.V., Best, the Netherlands) was used in combination with a C5-1 transducer, which is commonly used in abdominal and interventional procedures. The probe was placed on the right upper quadrant of the abdomen, left of and right under the rib cage. Coupling gel was applied between the skin and abdominal phantom, and the skin and ultrasound probe. The insertion angle with respect to the ultrasound probe was approximately 30° and 45°. The needle was captured in-plane.

## Results

### Liver and rib cage motion analysis

#### Liver and rib motion

**Fig. 6:**
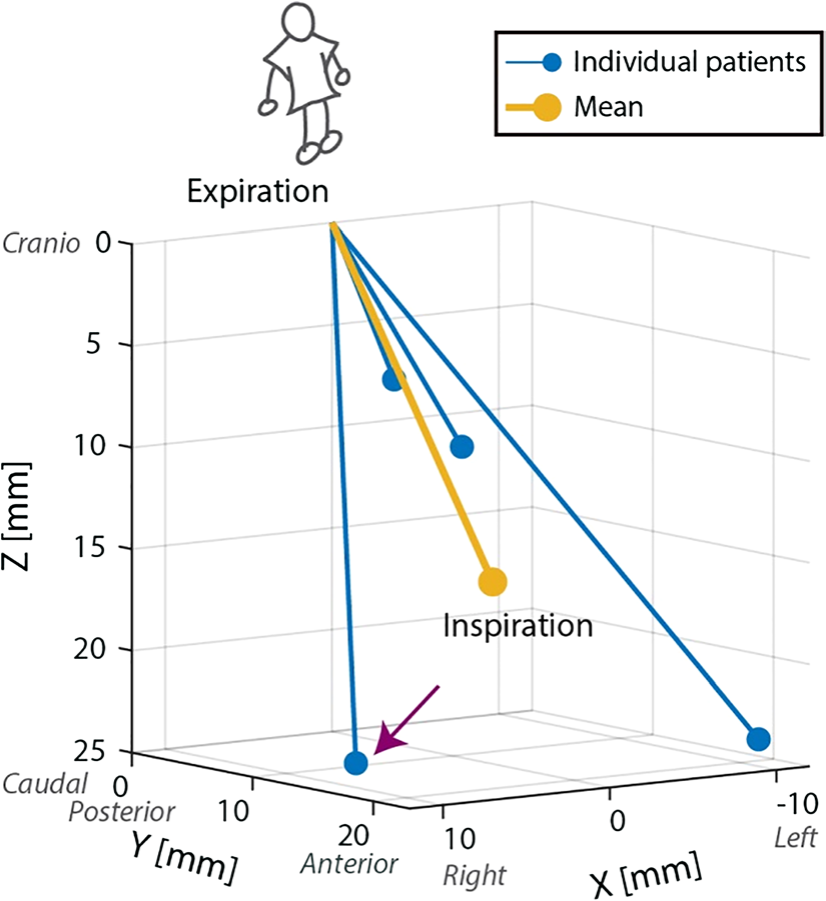
Motion patterns of the liver from expiration to inspiration (*n* = 4). Translational motion is predominantly toward the right (2 mm), anterior (15 mm) and caudal (16 mm) direction

The results of the motion analysis can be seen in Fig. 6. This figure shows the movement of the livers subjected to breathing from expiration toward inspiration in all directions, being: left–right (X), posterior-anterior (Y) and cranio-caudal (Z). From the state of expiration to inspiration, the centroids of the livers moved predominantly toward the right (mean: 2 mm, range: − 11 to 11 mm), anterior (mean: 15 mm, range: 9–21 mm) and caudal (mean: 16 mm, range: 6–24 mm) direction. Note that during inspiration the liver can move to either left or right, depending on the patient. The mean total stroke was 22 mm (range: 11–33 mm).

The seventh rib of the patients moved also predominantly toward the right (mean: 14 mm, range: 7–21 mm), anterior (mean: 7 mm, range: − 3 to 18 mm) and caudal (mean: 1 mm, range: − 11 to 10 mm) direction. Although the direction of rib motion coincides with liver motion, its magnitude differs. The mean total stroke was 20 mm (range: 14–30 mm).

#### Error estimation patient movement

The ninth vertebra of the patients moved predominantly to the right (mean: 0.5 mm, range: − 0.6 to 2.1 mm), anterior (mean: 1.5 mm, range: − 0.3 to 2.8 mm) and caudal direction (mean: 0.2 mm, range: − 0.8 to 0.1 mm) from the state of expiration to inspiration. Total movement of the individual vertebras during breathing was on average 1.6 mm, with a minimum of 0.4 mm and a maximum of 2.8 mm.

### Motion validation of the liver phantom

The motion vector and anthropomorphics of the patient indicated with the purple arrow in Fig. 6 were chosen as an input for the liver phantom. We chose this patient as it has the largest total stroke toward the right (total stroke: 31 mm, right: 11 mm, anterior: 17 mm, caudal: 24 mm). Based on the signal from the reference sensor, the combined standard uncertainty was 0.10 mm with the motor of the linear stage turned on, and 0.07 mm with the motor turned off. There is more electromagnetic interference when the linear stage was turned on. We did not subtract these noise levels from the final measurements, as they are negligibly small.

**Fig. 7:**
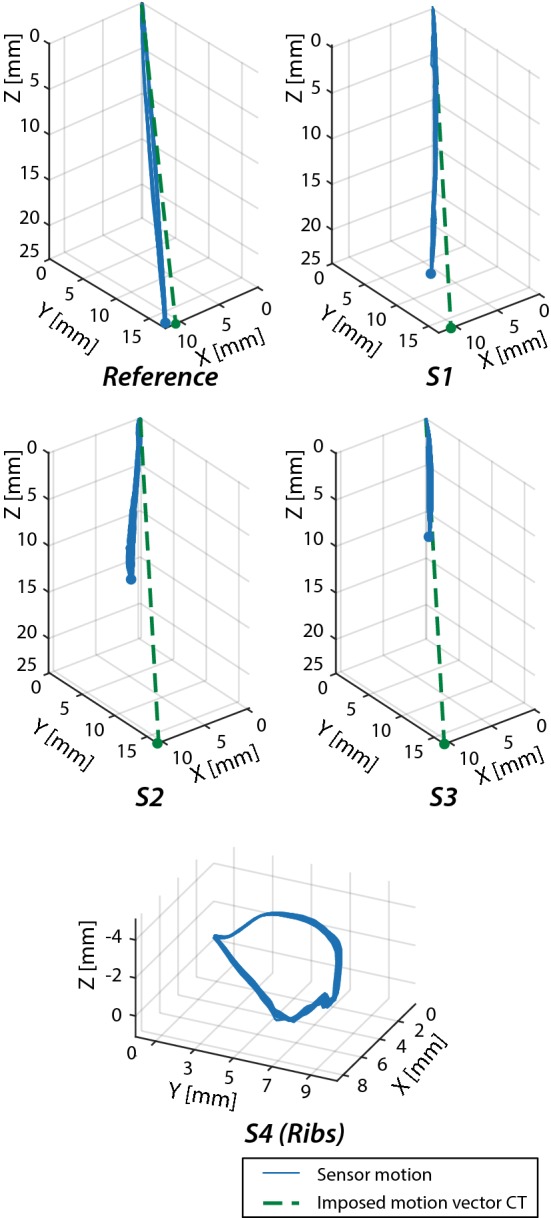
Liver and rib phantom movement during expiration and inspiration (three trials of 12 Hz for 60 s)

Figure 7 shows the results of the motion validation of the liver phantom for the three sensors on the PVA liver and the reference sensor on top of the stage. The amplitude and direction of the reference sensor are comparable to the imposed motion vector from the CT scans (green dotted line). The direction for all PVA liver sensors (S1 to S3) is comparable to the imposed motion vector, whereas the amplitude decreases for S2 and S3 compared to the real situation. This resulted in larger deformation of the PVA liver than the patient’s liver, probably caused by the limited space between the caudal end of the PVA liver and the plastic end plate.

Corresponding rib motion in the patient had a total stroke of 30 mm (right: 21 mm, anterior: 18 mm, caudal: 9 mm). Compliant rib motion phantom can be seen in Fig. 7-S4 (ribs). It has a circular pattern and a total stroke of 13 mm (right: 8 mm, anterior: 10 mm, cranio: 3 mm). This means that the movement of the ribs in right–left and anterior–posterior directions corresponds with the real situation as opposed to cranio-caudal direction.

**Fig. 8:**
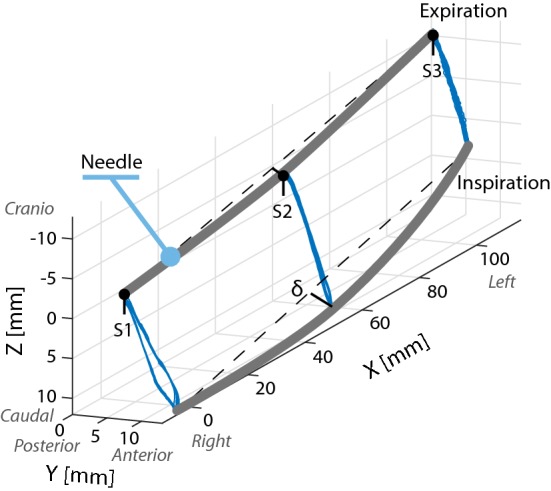
Ablation needle movement during expiration and inspiration state of the liver phantom (measured at 12 Hz for 60 s). *δ* = Needle deflection

Needle deflection from the state of expiration to inspiration is shown in Fig. 8. Upon insertion, i.e., the state of expiration, mean needle deflection (*δ*) was 1.6 mm, whereas the mean deflection increased to 3.8 mm during inspiration. The sensors did not change position with respect to the needle itself during the measurements, as there was almost no difference within the expiration and inspiration positions during the 60-s trial (standard deviations ranging from 0.1 to 0.7 mm).

### Needle visibility: ultrasound

**Fig. 9:**
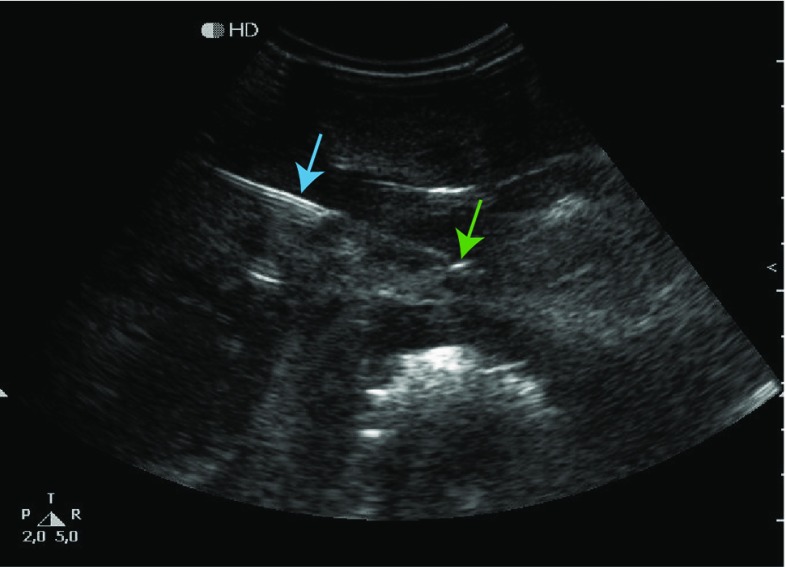
Ultrasound image of the liver phantom indicating needle shaft (blue arrow) and needle tip (green arrow) (gain = 23)

Figure 9 shows the result of the needle visibility validation. The needle shaft and the needle tip can be identified on the ultrasound picture (in-plane).

## Discussion

This study presents a design and validation of a liver phantom to be used for needle-based interventions. We fabricated a heterogeneous, ultrasound compatible phantom material, PVA, based on CT image segmentations of a real patient, and we simulated breathing motion. We validated the phantom motion of one patient by using an electromagnetic tracking system and compared the results with the CT images. In addition, we compared needle deflection in the phantom with those found in thermal ablation procedures of liver tumors, as previously described in the literature [2].

This research studied the motion of the liver and rib cage subjected to respiration as observed in four patients, by comparing the liver centroids during inspiration and expiration. All patients’ livers moved to caudal and anterior direction from the state of expiration to inspiration. However, for different patients they moved to either left or right. These results are in agreement with those obtained by Tsai et al. [25]. Presumably, this is caused by a difference in breathing type of the patients: diaphragmatic or chest.

The mean total liver stroke, based on the CT scans of the patients that were included in our study, was found to be 22 mm. Previous studies on liver motion show a total stroke ranging from a couple of mm up to a couple of cm (e.g., [25–30]). Our results are within this range, despite being on the higher end of the spectrum. This might be explained by the fact that the patients in our study were specifically asked to perform deep inspiration and maximum expiration, causing larger displacements than during ordinary breathing. As our liver phantom allows for adjustments of its motion vector, in terms of magnitude and direction, we do not foresee any problems in applying different motion patterns.

The motion stage uses one of the aforementioned motion vectors of patients as an input to simulate breathing in our phantom. For the liver, we found comparable motion patterns in terms of magnitude and direction for the superior parts of the liver, whereas the magnitude decreased for the inferior parts of the liver, i.e., the sensors that were placed further away from the applied motion (Fig. 7). This resulted in an observed deformation several mm bigger than in the corresponding patient. However, this is not particularly surprising given the fact that the PVA-supporting abdomen is mechanically not exactly the same as compared to the real situation in which the abdomen consists of several different organs and tissue types. If the exact same motion is preferred, more research is needed into the mechanical characteristics of the abdominal organs to correctly mimic the abdomen using PVA-based phantoms. Nevertheless, we believe that the current mimicked motion is sufficient for the purpose of novel instrument validation, as the phantom, in addition to the aforementioned similarities, also shows relative motion between the liver and rib cage, which is one of the factors that contribute to needle deflection. Future liver phantoms could include a layer of phantom muscle tissue that presumably has an influence on needle insertion forces and deflection too.

The most important clinically relevant finding of this research concerns the comparison between needle deflection in our phantom and the real situation. Unwanted bending of the needle upon insertion is a problem in interventional radiology and complicates precise placement [1]. We compared the needle deflection with the deflection quantified using CT scans of in vivo procedures of tumor ablations, reported in [2]. Bending was defined in the same manner as in the present study and ranged from 0 to 7 mm, which is in the same order of magnitude as observed in our liver phantom.

Although it is possible to make patient-specific phantoms with the current methods, the PVA phantom production is time-consuming. Several freeze–thaw cycles are needed to reach the desired amount of heterogeneity for it to be comparable to real tissue. Future developments in 3D printing of soft materials might overcome this limitation. For instance, Tan et al. [31] studied super-soft 3D-printed hydrogels, matching the stiffness of brain and lung tissue.

We emphasize three advantages of a tissue-mimicking material such as PVA, over the use of biological tissue. In general, tissue-mimicking materials (1) are durable, (2) are tunable and (3) can easily be used without violating ethical and regulatory guidelines. Tunability is especially important in case of future additional features of liver phantoms, such as mimicking diseased tissue and including target lesions. The third advantage is crucial for testing early prototypes of medical instruments developed by technical research groups, who often do not have easy access to an experimental laboratory with a permit to handle biological tissue.

Besides its purpose as a validation setup for novel needles, our liver phantom has the potential to be used as a trainings tool for interventional radiologists. In that specific case, we propose further research by performing user experience tests and extensive ultrasound compatibility experiments. Although we did not focus on the exact match between ultrasound of real livers and our phantom, we highlighted (Fig. 9) that all important structures are visible, which is sufficient for novel instrument validation. Ultrasound imaging of the phantom could, e.g., be improved by applying coupling gel between the PVA liver, PVA abdomen and the silicone skin phantom and by the addition of glass beads to induce more realistic acoustic backscattering [32].

As a final note, the gathered motion data of this research cannot only be used for designing physical liver phantoms, but are applicable for a broader scope, for example for the development of virtual simulators [33]. Therefore, the 3D models of the livers and rib cage during inspiration and expiration have been made publicly available [24].

## Conclusion

In conclusion, the experiments showed that the developed anthropomorphic PVA liver phantom is in general able to simulate respiration-induced motion of liver tissue that can be found in patients. The magnitude of needle deflection in the liver phantom is comparable to real procedures and increases during breathing. The phantom can be used to validate novel instruments and/or robotic systems for needle-based interventions.

## Electronic supplementary material

Below is the link to the electronic supplementary material.

Supplementary material 1 (WMV 17603 kb)

## References

[1] de Jong TL, van de Berg NJ, Tas L, Moelker A, Dankelman J, van den Dobbelsteen JJ (2018). Needle placement errors: do we need steerable needles in interventional radiology?. Med Devices Evid Res (Auckland, NZ).

[2] de Jong TL, Klink C, Moelker A, Dankelman J, van den Dobbelsteen JJ (2018) Needle deflection in thermal ablation procedures of liver tumors: a CT image analysis. In: Medical Imaging 2018: image-guided procedures, Robotic Interventions, and Modeling, vol 10576, International Society for Optics and Photonics, p 105761L

[3] van de Berg NJ, Dankelman J, van den Dobbelsteen JJ (2017). Endpoint accuracy in manual control of a steerable needle. J Vasc Interv Radiol.

[4] Li P, Yang Z, Jiang S (2018). Tissue mimicking materials in image-guided needle-based interventions: a review. Mater Sci Eng C.

[5] Jiang S, Liu S, Feng W (2011). PVA hydrogel properties for biomedical application. J Mech Behav Biomed.

[6] de Jong TL, Pluymen LH, van Gerwen DJ, Kleinrensink G-J, Dankelman J, van den Dobbelsteen JJ (2017). PVA matches human liver in needle-tissue interaction. J Mech Behav Biomed.

[7] Nicholson R, Crofton M (1997). Training phantom for ultrasound guided biopsy. Br J Radiol.

[8] Li W, Belmont B, Shih A (2015). Design and manufacture of polyvinyl chloride (PVC) tissue mimicking material for needle insertion. Proc Manuf.

[9] Hungr N, Long JA, Beix V, Troccaz J (2012). A realistic deformable prostate phantom for multimodal imaging and needle-insertion procedures. Med Phys.

[10] Zell K, Sperl J, Vogel M, Niessner R, Haisch C (2007). Acoustical properties of selected tissue phantom materials for ultrasound imaging. Phys Med Biol.

[11] Cournane S, Cannon L, Browne JE, Fagan AJ (2010). Assessment of the accuracy of an ultrasound elastography liver scanning system using a PVA-cryogel phantom with optimal acoustic and mechanical properties. Phys Med Biol.

[12] Rethy A, Sæternes JO, Halgunset J, Mårvik R, Hofstad EF, Sánchez-Margallo JA, Langø T (2018). Anthropomorphic liver phantom with flow for multimodal image-guided liver therapy research and training. Int J Comput Assist Radiol Surg.

[13] Maneas E, Xia W, Nikitichev DI, Daher B, Manimaran M, Wong RY, Chang CW, Rahmani B, Capelli C, Schievano S, Burriesci G (2018). Anatomically realistic ultrasound phantoms using gel wax with 3D printed moulds. Phys Med Biol.

[14] Stewart HR, Bes EB (1968). Practical inferences from studies with a “breathing” liver phantom. Am J Roentgenol.

[15] Banovac Filip, Glossop Neil, Lindisch David, Tanaka Daigo, Levy Elliot, Cleary Kevin (2002). Liver Tumor Biopsy in a Respiring Phantom with the Assistance of a Novel Electromagnetic Navigation Device. Medical Image Computing and Computer-Assisted Intervention — MICCAI 2002.

[16] Cleary KR, Banovac F, Levy E, Tanaka D (2002) Development of a liver respiratory motion simulator to investigate magnetic tracking for abdominal interventions. In: Proceedings SPIE 4681, Medical Imaging 2002: Visualization, Image-Guided Procedures, and Display. 10.1117/12.466934

[17] Naghibi H, Costa PA, Abayazid M (2018) A soft robotic phantom to simulate the dynamic respiratory motion of human liver. In: 2018 7th IEEE international conference on biomedical robotics and biomechatronics (Biorob), 2018, IEEE, pp 577–582

[18] Hu Y, Zhou Y-K, Chen Y-X, Zeng Z-C (2017). Magnitude and influencing factors of respiration-induced liver motion during abdominal compression in patients with intrahepatic tumors. Radiat Oncol.

[19] Rohlfing T, Maurer CR, O’dell WG, Zhong J (2004). Modeling liver motion and deformation during the respiratory cycle using intensity-based nonrigid registration of gated MR images. Med Phys.

[20] Kikinis R, Pieper SD, Vosburgh KG, Jolesz F (2014). 3D Slicer: a platform for subject-specific image analysis, visualization, and clinical support. Intraoperative imaging and image-guided therapy.

[21] Zhu L, Kolesov I, Gao Y, Kikinis R, Tannenbaum A (2014) An effective interactive medical image segmentation method using fast growcut. In: MICCAI workshop on interactive medical image computing, 2014

[22] Cignoni P, Callieri M, Corsini M, Dellepiane M, Ganovelli F, Ranzuglia G (2008) MeshLab: an Open-source mesh processing tool. In: Sixth Eurographics Italian chapter conference

[23] Pietroni N, Tarini M, Cignoni P (2010). Almost isometric mesh parameterization through abstract domains. IEEE Trans Vis Comput Graph.

[24] de Jong TL, Moelker A, Dankelman J, van den Dobbelsteen JJ (2019) 3D models of liver and ribcage of four patients during inspiration and expiration. 4TU Centre for research data, dataset. 10.4121/uuid:1a6ebda2-dbba-4814-bdad-ba723632ea95

[25] Tsai Y-L, Wu C-J, Shaw S, Yu P-C, Nien H-H, Lui LT (2018). Quantitative analysis of respiration-induced motion of each liver segment with helical computed tomography and 4-dimensional computed tomography. Radiat Oncol.

[26] Hallman JL, Mori S, Sharp GC, Lu H-M, Hong TS, Chen GT (2012). A four-dimensional computed tomography analysis of multiorgan abdominal motion. Int J Radiat Oncol Biol Phys.

[27] Wysocka B, Kassam Z, Lockwood G, Brierley J, Dawson LA, Buckley CA, Jaffray D, Cummings B, Kim J, Wong R (2010). Interfraction and respiratory organ motion during conformal radiotherapy in gastric cancer. Int J Radiat Oncol Biol Phys.

[28] Bussels B, Goethals L, Feron M, Bielen D, Dymarkowski S, Suetens P, Haustermans K (2003). Respiration-induced movement of the upper abdominal organs: a pitfall for the three-dimensional conformal radiation treatment of pancreatic cancer. Radiother Oncol.

[29] Balter JM, Brock KK, Litzenberg DW, McShan DL, Lawrence TS, Ten Haken R, McGinn CJ, Lam KL, Dawson LA (2002). Daily targeting of intrahepatic tumors for radiotherapy. Int J Radiat Oncol Biol Phys.

[30] Brandner ED, Wu A, Chen H, Heron D, Kalnicki S, Komanduri K, Gerszten K, Burton S, Ahmed I, Shou Z (2006). Abdominal organ motion measured using 4D CT. Int J Radiat Oncol Biol Phys.

[31] Tan Z, Parisi C, Di Silvio L, Dini D, Forte AE (2017). Cryogenic 3D printing of super soft hydrogels. Sci Rep.

[32] Culjat MO, Goldenberg D, Tewari P, Singh RS (2010). A review of tissue substitutes for ultrasound imaging. Ultrasound Med Biol.

[33] Villard PF, Jacob M, Gould D, Bello F (2009). Haptic simulation of the liver with respiratory motion. Stud Health Technol Inform.

